# Anxiety Sensitivity and Intolerance of Uncertainty Uniquely Explain the Association of the Late Positive Potential With Generalized Anxiety Disorder Symptoms

**DOI:** 10.1111/psyp.70044

**Published:** 2025-04-03

**Authors:** Matt R. Judah, Hannah C. Hamrick, Benjamin Swanson, Morgan S. Middlebrooks, Grant S. Shields

**Affiliations:** ^1^ Department of Psychological Science University of Arkansas Fayetteville Arkansas USA

**Keywords:** anxiety sensitivity, generalized anxiety disorder, intolerance of uncertainty, late positive potential

## Abstract

Studies suggest that generalized anxiety disorder (GAD) symptoms are related to late positive potential (LPP) responses to negative images, suggesting greater attention. Anxiety sensitivity (AS) and intolerance of uncertainty (IU) are cognitive factors in GAD vulnerability that may be activated by negative stimuli, thereby explaining why the LPP and GAD symptoms are related. We examined whether AS and IU explain the association of the LPP with GAD symptoms. Eighty‐seven (77% women) young adults viewed 60 negative and 60 neutral images. The LPP was examined using both frequentist and Bayesian approaches. This revealed unique indirect effects of the LPP on GAD symptoms through AS and IU. Neither indirect effect was stronger, and the indirect effects were present regardless of using frequentist or Bayesian analyses or quantifying the LPP using residual‐based scores or difference scores. The indirect effects predicted not only GAD symptoms but social anxiety and depression as well, consistent with the role of AS and IU in transdiagnostic vulnerability. The findings support AS and IU as links that explain how attention to negative stimuli is related not only to GAD symptoms but to other internalizing symptoms as well.

## Impact Statement

The late positive potential is a neural indicator of attention that has linked attention to negative stimuli with generalized anxiety disorder (GAD). Our data show that this link is explained in part by two GAD risk factors: intolerance of uncertainty and fear of bodily symptoms of anxiety. The effects were robust to various analytic approaches.

Generalized anxiety disorder (GAD) is a condition characterized by excessive and uncontrollable worry that is associated with symptoms such as restlessness, irritability, and sleep disturbance (American Psychiatric Association 2022). GAD is among the most common psychological disorders (Kessler et al. 2012) with a lifetime prevalence of about 4% globally and 8% in the United States (Ruscio et al. 2017). Both incidence and prevalence are greatest during early adulthood (Beesdo et al. 2010; Terlizzi and Villarroel 2020). GAD is associated with lower quality of life, worse health, and functional impairment (Wilmer et al. 2021), and even subthreshold symptoms have been linked to impairment (Haller et al. 2014).

Theories of GAD describe heightened emotional reactivity (Mennin et al. 2002), which is supported by meta‐analytic data showing greater attention to negative stimuli in people with GAD (Goodwin et al. 2017). Attention to negative stimuli is thought to activate rigid and automatic schemas, including heightened perceptions of danger based on one's physiological arousal and the uncertainty of the situation (Behar et al. 2009; Carleton 2016; Clark and Beck 2010). Integrating components of several cognitive models, Roemer and Orsillo (2002), Roemer et al. (2005) proposed that GAD involves a fear of one's internal reactions to negative stimuli. As an example, they describe fear of anxious arousal, known as anxiety sensitivity that may be activated when stimuli trigger arousal. Complementary research has focused on distress reactions to situations that evoke uncertainty, known as intolerance of uncertainty (Dugas et al. 1998, 2005). Thus, GAD is thought to be driven not only by sensitivity to negative stimuli but also by reactions to the feelings that negative stimuli evoke.

Research supports a link between heightened attention to negative stimuli and GAD (Goodwin et al. 2017). This attention has been examined via brain activity while viewing negative images (e.g., depicting bodily mutilation). Viewing negative images evokes the late positive potential (LPP), an event‐related potential maximal at centroparietal electrodes 400–700 ms after stimulus onset (Cuthbert et al. 2000; Hajcak and Foti 2020). LPP amplitude reflects the modulation of attention by the motivational salience of a stimulus, being larger for salient stimuli, such as threatening images (Weinberg and Hajcak 2010). The LPP persists throughout the duration of image presentation (Hajcak and Olvet 2008) and is modulated by efforts to downregulate or upregulate emotional response, showing decreases or increases in amplitude, respectively (Moser et al. 2010). As an index of attention to negative stimuli, the LPP has been useful in testing theories that propose attention to negative stimuli as a factor in GAD.

Supporting cognitive theories, several studies suggest that individuals with GAD have a larger LPP response to negative images than to neutral images (MacNamara and Hajcak 2010; MacNamara et al. 2016), but others do not (Weinberg and Hajcak 2011). The association appears to be stronger during high working memory load (MacNamara and Proudfit 2014; White and Grant 2017), which can result from worry (Sari et al. 2017). The arrangement of stimuli into blocks may also be important. Worry has been linked to a larger difference between the LPP responses to negative and neutral images when negative and neutral images are presented in separate blocks (Burkhouse et al. 2015). However, worry has been associated with a smaller difference when blocks contain both negative and neutral stimuli (Grant et al. 2015, 2023; but see Weinberg and Hajcak 2011). Although the relation of the LPP to GAD and worry is not clearly understood across studies, a greater difference in LPP response to negative compared to neutral images has been observed in several studies. This supports models that propose that GAD involves heightened attention to negative stimuli. However, there remains a need to identify cognitive processes that may explain the association between the LPP and GAD symptoms. Based on cognitive models, negative stimuli may not only grab attention and evoke emotion; they also may activate further reactions based on schemas that feelings of arousal and uncertainty portend danger.

The schema that arousal is dangerous is best represented by anxiety sensitivity (AS), which involves concerns about the consequences of anxious arousal (Reiss et al. 1986). AS plays a role in GAD vulnerability, predicting later symptom development (Allan et al. 2014). Limited data also suggest that AS is related to larger LPP responses to negative stimuli. Saulnier et al. (2021) found that AS was related to the LPP responses in an IAT task when anxiety words were paired with self‐words, but not when calm words were paired with self‐words. Two studies found AS to be related to larger LPP responses to images depicting anxious arousal (Allan et al. 2019; Hamrick et al. 2024). One study (Allan et al. 2019) also found the social concerns subdomain of AS to be positively correlated with a larger LPP response to negative images compared to neutral ones. These data, though limited, implicate AS as a factor in GAD that is linked to the modulation of attention to emotional images. Based on these findings and the theoretical description of AS‐based reactions to feelings triggered by negative stimuli (Roemer et al. 2005), there is a need to test whether AS explains the association between the LPP response to negative images and GAD symptoms.

Intolerance of uncertainty (IU) is another cognitive construct that plays a role in GAD vulnerability (Dugas et al. 2005; Saulnier et al. 2021; Shapiro et al. 2020). IU is a trait‐like low endurance of distress arising from ambiguity or lack of information (see Carleton 2016) that becomes activated in response to situations or stimuli in which outcomes are uncertain (Koerner and Dugas 2006). IU has been linked to maladaptive threat anticipation, learning, and processing in studies examining autonomic and neural measures (see Tanovic et al. 2018 for a review). Individuals with high IU exhibit greater reactivity to uncertainty in the anterior insula and amygdala (Tanovic et al. 2018), reduced threat extinction (Morriss 2019), and early attention to ambiguous stimuli (Zhou et al. 2023). Some studies have observed increased autonomic reactivity to unpredictable startle (Carsten et al. 2022), but these findings are mixed (Tanovic et al. 2018). Research also suggests that IU is related to greater attention to negative stimuli, even when they are predictable (Carsten et al. 2022; Del Popolo Cristaldi et al. 2021), possibly reflecting vigilance and preference for negative stimuli over ambiguous ones (Koerner and Dugas 2006). With respect to the LPP, most studies of IU have used simple stimuli conditioned to evoke a fear response (Bauer et al. 2020; Nelson et al. 2015). These studies found a larger LPP response to the conditioned stimulus compared to other similar stimuli, suggesting reduced fear generalization. A few studies have examined LPP responses to negative and neutral images following a cue used to manipulate the certainty of the subsequent image valence. These studies observed a larger LPP response to negative images, but one found that this was reduced when stimuli were unpredictable (Gole et al. 2012) while another did not find an effect of predictability (Wiese et al. 2023). Overall, the pattern of findings resembles what has been seen in the relation between the LPP and GAD: greater LPP response to negative compared to neutral stimuli. Like AS, theoretical work has described IU as a dispositional trait that may be activated by stimuli and maintain GAD symptoms (Koerner and Dugas 2006).

The present study examined whether the association between the LPP and GAD symptoms was explained by AS and IU using a mediation model to test indirect effects and compare them. This was based on theories (Clark and Beck 2010; Koerner and Dugas 2006; Roemer et al. 2005) that describe GAD as involving attention to negative stimuli, reflected in the LPP response to negative stimuli, which activate threat schemas that include AS and IU. We hypothesized indirect effects through both AS and IU. Analyses were conducted on the LPP residual‐based score (i.e., unstandardized residuals from a regression in which the neutral LPP predicted the negative LPP; Meyer et al. 2016) and on the LPP difference score (i.e., LPP response to negative minus LPP response to neutral; see Supporting Information S1). Both frequentist and Bayesian approaches were examined. Because AS and IU are transdiagnostic processes broadly associated with internalizing disorders and to determine whether findings were specific to GAD symptoms, we also tested effects on social anxiety and depression (see Supporting Information S1). We also used a sensitivity analysis based on Georgeson et al. (2023) method to estimate the main model's indirect effects at varying autoregressive and cross‐lagged correlations in a hypothetical longitudinal design (see Supporting Information S1). Our goal in using cross‐sectional mediation was to test whether AS and IU statistically explain the association between the LPP and GAD symptoms, not to test causal mediation, which would require a longitudinal design. Although the sensitivity analysis cannot overcome the limitations of cross‐sectional design to establish causal mediation, we included this analysis to inform the viability of future longitudinal studies.

## 1 Methods

### 1.1 Participants

Eighty‐seven undergraduate students from a large southern university completed the study after being recruited from a psychology program research participation pool. We determined, post hoc, that the sample size exceeded the minimum of 71 participants needed to achieve power of 0.80 to detect an indirect effect with medium effect sizes in the *a* and *b* paths based on simulations conducted by Fritz and MacKinnon (2007) on models using bias‐correct bootstrapping. Participants were compensated with research credit for courses. Inclusion criteria were being at least 18 years old and able to read English. The mean age was 20.22 years (SD = 3.72), and participants primarily identified as cisgender women (77.0%) and White (70.1%). Demographic data are displayed in Table 1.

**TABLE 1:** Sample demographic information.

	*n* (*M*)	% (SD)
Age	(20.22)	(3.72)
Gender		
Cisgender woman	67	77.0
Cisgender man	20	23.0
Transgender or gender nonbinary	0	0
Ethnicity^a^		
White	61	70.1
Latinx	18	20.7
Black	13	14.9
East Asian	9	10.3
South Asian	4	4.6
Native American/American Indian	2	2.3
Pacific Islander	1	1.1

*Note: N* = 87.

^a^ Groups are not mutually exclusive.

### 1.2 Measures

#### 1.2.1 Anxiety Sensitivity

AS was assessed using an 18‐item self‐report scale, the Anxiety Sensitivity Index‐3 (ASI‐3; Taylor et al. 2007). Agreement with each item (e.g., “it scares me when my heart beats rapidly”) was rated on a Likert scale from 0 (very little) to 4 (very much), such that higher sum scores across items indicates greater AS. Internal consistency was excellent, *α* = 0.91.

#### 1.2.2 Generalized Anxiety Symptoms

Generalized anxiety disorder symptoms were assessed using the Generalized Anxiety Disorder‐7 Scale (GAD‐7; Spitzer et al. 2006). Symptoms of GAD were assessed using the 7‐item self‐report GAD‐7, a scale that has been validated in psychiatric (Spitzer et al. 2006) and undergraduate (White and Karr 2023) samples. The frequency of symptoms (e.g., “feeling nervous, anxious or on edge”) over the past two weeks was rated on a Likert scale from 0 (not at all) to 3 (nearly every day), such that a higher sum score across items indicated higher GAD symptom frequency. Internal consistency was good, *α* = 0.86. Twenty‐eight participants (32.2%) scored at or above the provisional diagnostic cut score of 10, a rate similar to other post‐pandemic studies (White and Karr 2023).

#### 1.2.3 Intolerance of Uncertainty

IU was assessed using the Intolerance of Uncertainty Scale‐12 (IUS‐12; Carleton et al. 2007), which consists of 12 items. Each item (e.g., “unforeseen events upset me greatly”) was rated using a 5‐point Likert scale that ranges from 1 (not at all characteristic of me) to 5 (entirely characteristic of me), such that a higher sum score across items indicated higher IU. Internal consistency was good, *α* = 0.87.

#### 1.2.4 Social Anxiety

Social anxiety was measured using the 6‐item Social Interaction Anxiety Scale (SIAS‐6; Peters et al. 2012). Items assessing anxiety in social situations are rated on a scale from 0 (not at all characteristic or true of me) to 4 (extremely characteristic or true of me). A sum total score was calculated such that social anxiety was indicated by higher scores. Internal consistency was good, *α* = 0.84.

#### 1.2.5 Depression

Depression symptoms were measured using the 9‐item Patient Health Questionnaire (PHQ‐9; Spitzer et al. 1999). Items assess the frequency of depression symptoms rated from 0 (not at all) to 3 (nearly every day). A sum score was calculated such that higher scores indicated greater severity of depression symptoms. Internal consistency was good, *α* = 0.81.

### 1.3 Procedure

All procedures were reviewed and approved by the university's institutional review board. Participants provided informed consent and completed the self‐report measures on a desktop computer in a lab setting while being inconspicuously monitored by research assistants in an adjacent room. Electrodes were applied to the scalp using a custom 33‐channel EEG cap manufactured by BioSemi. An electrode was applied to each mastoid, below the left eye, and lateral to the outer canthus of each eye. The stimulus display monitor (Dell S2716DG, 27.0‐in. 60 Hz) was positioned at a viewing distance of 70 cm. Stimuli were presented at 1024 × 768 resolution using Presentation (v. 19.0; Neurobehavioral Systems Inc., Berkley, CA, www.neurolabs.com).

Participants completed a passive viewing task in which they viewed randomized blocks, each consisting of 20 randomized images. Each block contained images of negative valence (60 total) or neutral valence (60 total) selected from the international affective picture system (IAPS; Lang et al. 2008)^1^. Negative images had more negative valence ratings (*M* = 2.19, SD *= 0*.37) compared to neutral images (*M* = 5.08, SD = 0.57, *t* = 32.98, *p* < 0.001) and higher arousal ratings (*M* = 6.05, SD = 0.62) compared to neutral images (*M* = 3.19, SD = 0.43, *t* = 29.19, *p* < 0.001). Each image subtended a visual angle of 25.36 × 19.06 and was presented on a gray (RGB: 192, 192, 192) background. Images were preceded by a central fixation cross lasting 500 ms. After this, the image was presented for 2000 ms. The intertrial interval varied randomly between 800 ms, 1000 ms, or 1200 ms. Participants were instructed to look at the fixation cross when it appeared and to look at each image.

### 1.4 EEG Processing

EEG data were recorded at 1024 Hz using a BioSemi ActiveTwo system with 33 channels positioned according to the 10–20 system. Eye movements and blinks were recorded with additional electrodes placed below the left eye and lateral to the outer canthus of each eye. Offline processing was done in EEGLAB (v2022.0; Delorme and Makeig 2004) and ERPLAB (v.8.30; Lopez‐Calderon and Luck 2004). Data were referenced offline to the average of the mastoids and filtered using a 0.1 Hz high‐pass filter (see Taner et al. 2015). Ocular artifacts (i.e., blinks and saccades) were identified using independent components analysis and were removed. Bad channels were identified by visual inspection and interpolated. The data were segmented from −200 to 2000 ms relative to stimulus onset and baseline‐corrected to the pre‐stimulus interval. Automated routines in ERPLAB were used for artifact detection. Trials were excluded if they contained an eye blink within 200 ms of stimulus onset because a blink overlapping stimulus onset could delay the sensation of the stimulus. Trials also were excluded if any sample exceeded ±200 μV relative to the baseline. A routine to detect flatlining electrodes did not find any instances of flatlining. As has been done by other research groups, the LPP was measured at the Pz electrode (McGhie et al. 2021; Weinberg et al. 2021). Based on measurement in previous studies (Hill et al. 2023; Kraft et al. 2022; Zhang et al. 2021), including psychometric research (Moran et al. 2013), mean amplitude ERPs were measured from 400 to 700 ms, 700 to 1000 ms, and 1000 to 2000 ms post‐stimulus onset after subtracting the mean of the baseline period.

#### 1.4.1 ERP Data Quality and Reliability

Mean amplitude reliabilities were estimated using standard measurement errors ($\hat{\text{SMEs}}$), which are estimates of the standard deviation of each participant's sampling distribution (Luck et al. 2022). From 400 to 700 ms, the ratio of the root mean square of the $\hat{\mathrm{SME}}$, i.e., RMS ($\hat{\mathrm{SME}}$), to the SD was small in the case of negative, 0.38, and neutral trials, 0.44, suggesting that measurement error contributed little to observed amplitude. It was similarly small from 700 to 1000 ms (negative = 0.46; neutral = 0.63). However, it was large from 1000 to 2000 ms (negative = 1.59; neutral = 1.92). Based on reliability as true score variance divided by total variance, reliability was estimated as $\hat{\text{Reliability}}=\frac{{\hat{\mathrm{VAR}}}_{\text{Total}}-\mathrm{MS}\left(\hat{\mathrm{SME}}\right)}{{\hat{\mathrm{VAR}}}_{\text{Total}}}$, where MS ($\hat{\mathrm{SME}}$) is the Mean Square $\hat{\mathrm{SME}}$, which is RMS ($\hat{\mathrm{SME}}$)^2^. From 400 to 700 ms, reliability was good for negative trials, $0.86=\frac{30.025-4.246}{30.025}$, and neutral trials, $0.81=\frac{20.411-3.950}{20.411}.$ From 700–1000 ms, reliability was acceptable for negative trials, $0.79=\frac{23.195-4.985}{23.195}$, but poor for neutral trials, $0.60=\frac{12.265-4.878}{12.265}.$ Reliability was poor in the 1000–2000 ms window for both threat, $0.61=\frac{16.344-6.416}{16.344}$, and neutral trials, $0.60=\frac{10.806-6.321}{10.806}$. Using the odd and even trials, split‐half reliabilities of the residual‐based LPP and difference score LPP were estimated using the Spearman–Brown prophecy formula. Reliability was acceptable for the residual‐based score, *r* = 0.70, but poor for the difference score, *r* = 0.26.

### 1.5 Analytic Approach

**FIGURE 1:**
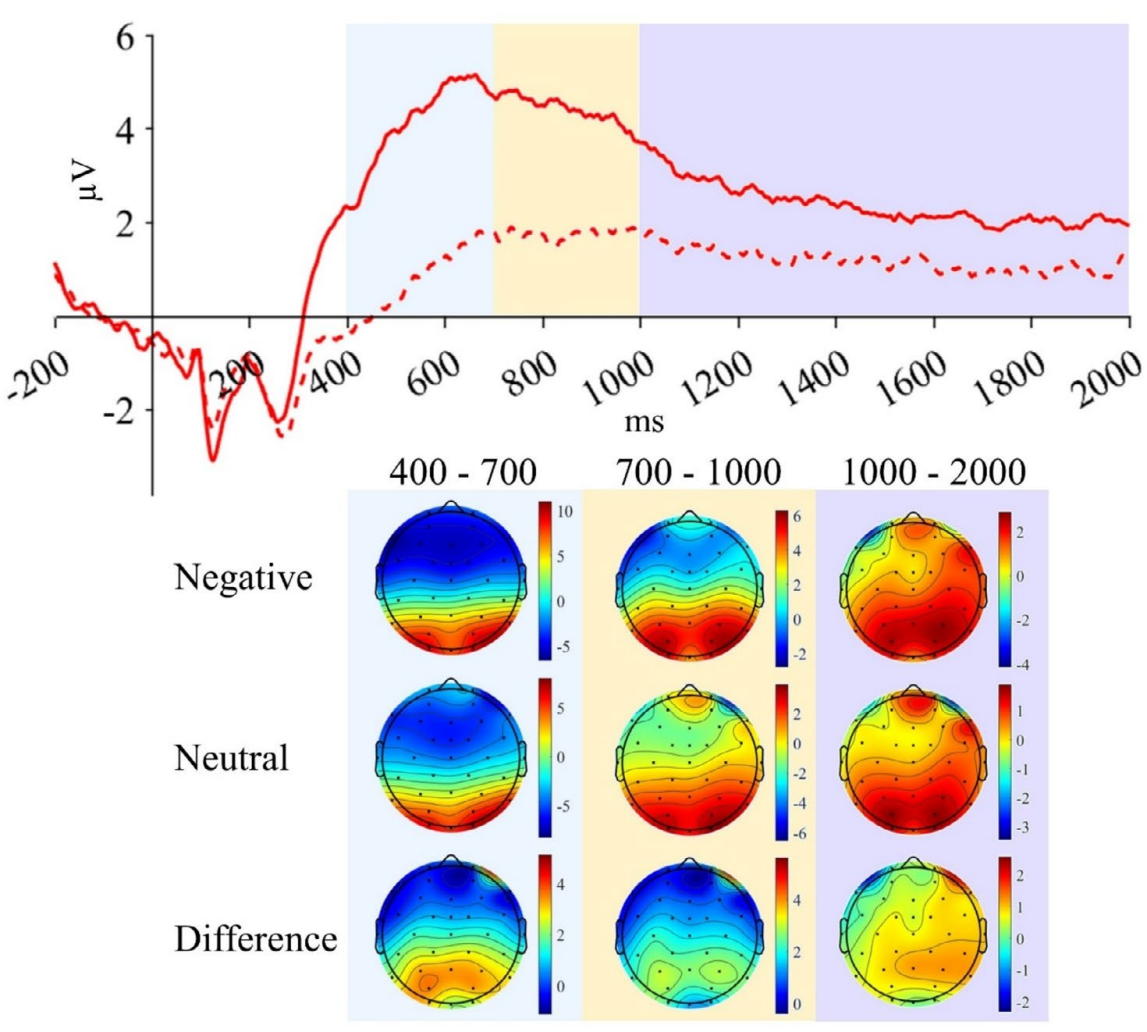
ERP response locked to the onset of negative (solid) and neutral (dotted) images.

Analyses were conducted on mean amplitudes from 400 to 700 ms based on acceptable reliability estimates (see Figure 1). Due to the poor reliability within the 700–1000 ms and 1000–2000 ms windows, analyses of these windows were included in the Supporting Information S1 only. Bivariate correlations between study variables are reported in Table 2. All variables were normally distributed^2^. The Breusch–Pagan test supported the assumption of homoscedasticity. A parallel mediation analysis was conducted using the PROCESS macro (version 4.1; Hayes 2022) to examine the indirect effect of the residual‐based LPP (i.e., residuals of a regression in which LPP response to neutral images predicted LPP response to negative images; see Meyer et al. 2016) on GAD symptom severity through AS and IU. A 95% bias‐corrected confidence interval, based on 5000 bootstrapped samples, was used to estimate standardized coefficients. The analyses were repeated using the difference score LPP (i.e., mean amplitude of negative trials minus mean amplitude of neutral trials). The results are included in the Supporting Information S1, and the direction and significance of all effects were the same in both analyses. The Supporting Information S1 also contains analyses testing the indirect effects of the difference LPP on social anxiety and depression through AS and IU. This was done to test the specificity of the findings to GAD symptoms versus internalizing symptoms more broadly.

**TABLE 2:** Means, standard deviations, and correlations between study variables.

	1	2	3	4	5	6	7
1. Residual LPP	—						
2. Difference LPP	0.997***	—					
3. ASI‐3	0.230*	0.240*	—				
4. IUS‐12	0.241*	0.235*	0.492***	—			
5. GAD‐7	0.297**	0.289**	0.557***	0.528***	—		
6. SIAS‐6	0.185	0.177	0.544***	0.560***	0.420***	—	
5. PHQ‐9	0.251*	0.256*	0.572***	0.558***	0.723***	0.558***	—
*M*	0.000	3.690	18.632	31.897	7.264	6.057	7.931
SD	3.439	3.448	13.516	8.849	5.003	5.371	5.730

*Note:* ***p < 0.001; **p < 0.01; *p < 0.05 (two‐tailed).

We also conducted a Bayesian parallel mediation analysis in Julia, v1.9.3, with the Turing package, v0.29.3, using the No U‐Turn Sampler (NUTS) Hamiltonian Monte Carlo (HMC) method. Bayesian parameter estimation was conducted using four chains, each of which consisted of 12,500 iterations, inclusive of 2500 burn‐in iterations. All variables were first standardized. The Bayesian model was then as follows:

β_LPP to ASI_ ~ *N*(0, 1) [−1, 1].

β_LPP to IUS_ ~ *N*(0, 1) [−1, 1].

β_LPP to GAD‐7_ ~ *N*(0, 1) [−1, 1].

β_ASI to GAD‐7_ ~ *N*(0, 1) [−1, 1].

β_IUS to GAD‐7_ ~ *N*(0, 1) [−1, 1].

σ_ASI_ ~ *N*(1, 1) [0, Inf].

σ_IUS_ ~ *N*(1, 1) [0, Inf].

σ_GAD‐7_ ~ *N*(1, 1) [0, Inf].

*E*_ASI *i*_ = LPP_*i*_ × β_LPP to ASI_.

ASI_*i*_ ~ *N*(*E*_ASI *i*_, σ_ASI_).

*E*_IUS *i*_ = LPP_*i*_ × β_LPP to IUS_.

IUS_*i*_ ~ *N*(*E*_IUS *i*_, σ_IUS_).

*E*_GAD‐7 *i*_ = LPP_*i*_ × β_LPP to GAD‐7_ + ASI_*i*_ × β_ASI to GAD‐7_ + IUS_*i*_ × β_IUS to GAD‐7_.

GAD‐7_*i*_ ~ *N*(*E*_GAD‐7 *i*_, σ_GAD‐7_).

All estimates achieved convergence, *R* ^^^s < 1.0001. In the Bayesian analysis section, 1‐*p*_*d*_ indicates the Bayesian posterior probability of observing a value of zero or with the opposite sign as the mean posterior estimate for that parameter (e.g., the proportion of the posterior distribution that is zero or positive when the mean estimate is negative). Therefore, 1‐*p*_*d*_ roughly corresponds to a Bayesian one‐tailed *p* value.

Cross‐sectional mediation models have been found to produce biased parameter estimates when compared to longitudinal mediation models because they do not model the stability (i.e., autoregressive correlation) and cross‐lags of the mediator and outcome variables (Maxwell and Cole 2007; Maxwell et al. 2011). Although establishing causal mediation was not our goal, nor is it possible in a cross‐sectional study, we conducted a sensitivity analysis based on the method of Georgeson et al. (2023; see Supporting Information S1) to inform whether a future longitudinal study would be viable.

## 2 Results

### 2.1 Frequentist Mediation Analysis

**FIGURE 2:**
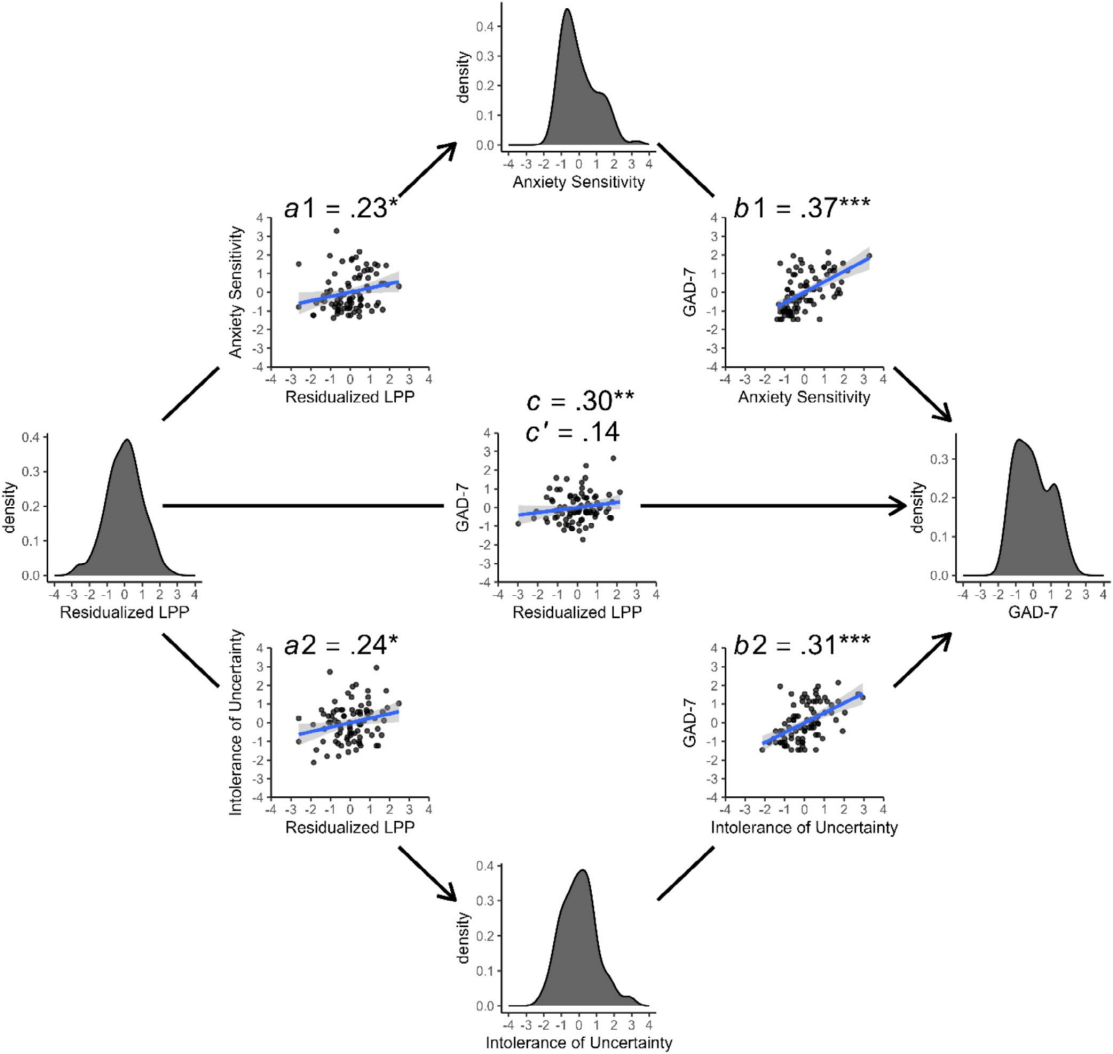
Indirect effects of the residual‐based LPP on GAD‐7 symptoms through anxiety sensitivity and intolerance of uncertainty. Scatter plots with GAD‐7 are modeled residuals when appropriate.

The residual‐based LPP from 400 to 700 ms was associated with greater AS, *a1* = 0.23 [0.02, 0.44], *t* = 2.18, and greater IU, *a2* = 0.24 [0.03, 0.45], *t* = 2.29. Higher AS predicted higher GAD‐7 scores, *b1* = 0.37 [0.18, 0.57], *t* = 3.82. Higher IU also predicted higher GAD‐7 scores, *b2* = 0.31 [0.12, 0.51], *t* = 3.19. There was an indirect effect of the LPP on GAD‐7 scores through AS (*a1b1* = 0.09 [0.01, 0.19]) and through IU (*a2b2* = 0.08 [0.01, 0.17]). The indirect effects contrast, 0.01 [−0.09, 0.12], showed that the indirect effects did not differ. The total effect, *c* = 0.30 [0.09, 0.51], accounted for 8.84% of the variance in GAD‐7 scores. There was no direct effect of the LPP on GAD‐7 scores, *c'* = 0.14 [−0.04, 0.31]. These effects are displayed in Figure 2.

### 2.2 Bayesian Mediation Analysis

**FIGURE 3:**
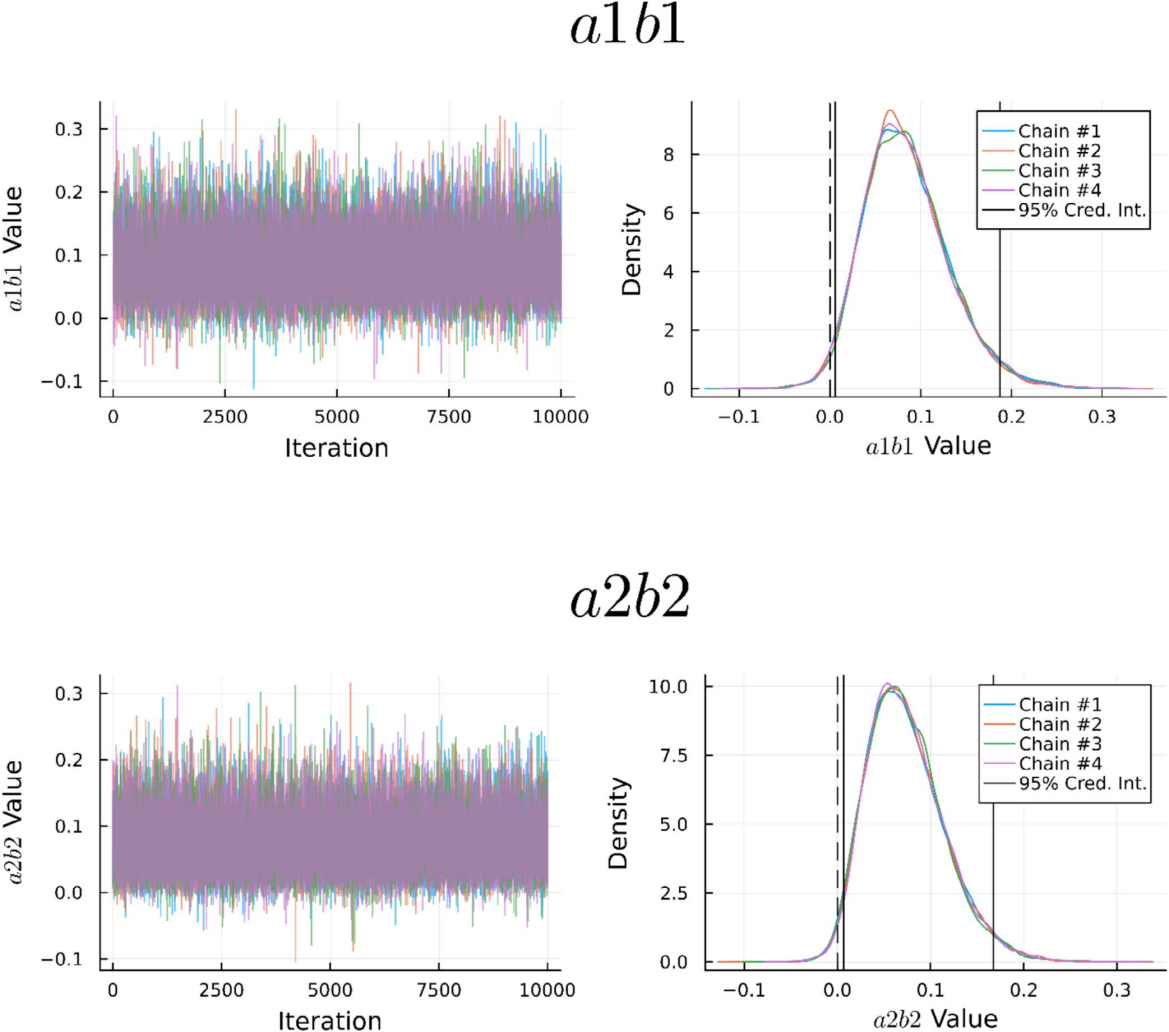
Bayesian estimates of the indirect effects of the residual‐based LPP through anxiety sensitivity (a1b1) and intolerance of uncertainty (a2b2).

The results of the Bayesian parallel mediation analysis were similar to the frequentist analyses. The residual‐based LPP was associated with greater AS, *a1* = 0.23, 95% credible interval: [0.02, 0.44], 1‐*p*_*d*_ = 0.016, and greater IU, *a2* = 0.24, 95% credible interval: [0.03, 0.45], 1‐*p*_*d*_ = 0.012. Higher AS predicted higher GAD‐7 scores, *b1* = 0.37, 95% credible interval: [0.18, 0.56], 1‐*p*_*d*_ < 0.001. Higher IU also predicted higher GAD‐7 scores, *b2* = 0.31, 95% credible interval: [0.12, 0.50], 1‐*p*_*d*_ = 0.001. There was an indirect effect of the residual‐based LPP on GAD‐7 scores through AS (*a1b1* = 0.08, 95% credible interval: [0.01, 0.19], 1‐*p*_*d*_ = 0.016) and through IU (*a2b2* = 0.07, 95% credible interval: [0.01, 0.17], 1‐*p*_*d*_ = 0.013; see Figure 3). The indirect effects contrast, 0.01, 95% credible interval [−0.12, 0.14], 1‐*p*_*d*_ = 0.436, showed that the indirect effects did not differ; 87.0% of posterior samples fell within a region of practical equivalence (i.e., |sampled value| < 0.10, which is less than a small effect). The total effect, *c* = 0.29 [0.10, 0.49], 1‐*p*_*d*_ = 0.002, accounted for 8.69% of the variance in GAD‐7 scores. There was no direct effect of the residual‐based LPP on GAD‐7 scores, *c'* = 0.13, [−0.03, 0.31], 1‐*p*_*d*_ = 0.06, though it should be noted that only 33.5% of posterior samples fell within a region of practical equivalence (i.e., |sampled value| < 0.10).

## 3 Discussion

As hypothesized, we found unique indirect effects of the LPP on GAD symptoms through AS and IU. The findings support the positive association between the LPP response to negative images and GAD symptoms observed in prior studies (MacNamara et al. 2016). Some studies have not observed this association, with findings possibly suggesting increased LPP response to neutral images (Weinberg and Hajcak 2011), and divergent findings may be due to moderators (Botelho et al. 2023; MacNamara and Hajcak 2010; MacNamara and Proudfit 2014). Our findings also diverge from a few studies of worry, the core feature of GAD, which were associated with a smaller difference between LPP responses to negative and neutral images (Grant et al. 2015, 2023; Kausche et al. 2022). Most of these studies used mixed blocks that included both negative and neutral images, which may increase the salience of neutral images because negative stimuli are unpredictable and because neutral images also signal the absence of what might have been a negative image. Alternatively, it is possible that subdomains of anxiety, namely anxious arousal (i.e., somatic symptoms) and anxious apprehension (i.e., worry), both of which are implicated in GAD and other anxiety disorders, have divergent associations with LPP responses. A recent study found that anxious apprehension was associated with a blunted LPP response to negative images, but anxious arousal was not (Kausche et al. 2022). Although more research is needed to resolve this, there is evidence that other neural markers relate differently to worry and somatic anxiety. For example, Härpfer et al. (2021) found reduced right frontal resting alpha was related to worry, but not somatic anxiety. In short, although the association between the LPP to negative (vs. neutral) images and GAD may depend upon specific factors, our results nonetheless support the existence of this association.

Consistent with the role of AS and IU as transdiagnostic processes across internalizing disorders, we found indirect effects of the LPP on social anxiety and depression through AS and IU (see Supporting Information S1). Notably, the LPP was correlated with GAD symptoms at the zero‐order level, but not with social anxiety or depression. Most research examining the LPP response to negative and neutral images has tested associations with GAD symptoms or worry, but there are increasingly more studies linking the LPP to social anxiety and depression. MacNamara et al. (2019) found the difference LPP (negative minus neutral) to be positively related to social anxiety. Similarly, Kinney et al. (2019) found a larger LPP response to negative images in socially anxious individuals, but this was only in a late window (i.e., 5000‐7000 ms after stimulus onset). With respect to depression, some studies have reported a blunting of the LPP such that the negative‐minus‐neutral difference is negatively related to depression (MacNamara et al. 2016). In contrast, other studies found the LPP to be positively related to depression (Zhang et al. 2016) while others found no association (Nikolin et al. 2022). In our findings, there was no evidence of zero‐order correlations of the LPP with social anxiety or depression. However, the indirect effects of the LPP with social anxiety and depression reflect the complexity of emotional processing related to internalizing dimensions. Systematic testing of possible mechanisms and factors associated with resilience, vulnerability, and maintenance is needed.

We limited our main analyses to the 400–700 ms window because of poor reliability of the neutral LPP from 700 to 1000 ms and of the negative and neutral LPP from 1000–2000 ms (included in the Supporting Information S1). The pattern of findings in the 700–1000 ms window was similar to those in the early window, except they were qualitatively larger, and there was a direct effect between the LPP response and GAD symptoms. No indirect, direct, or total effects were evident in the 1000–2000 ms window. Although limited by low reliability, the later window analyses advance understanding of how the LPP over time relates to GAD symptoms. Time windows of 400–700 and 700–1000 ms are commonly examined (e.g., Dickey et al. 2021; Grant et al. 2023; Wang et al. 2020), but some studies have used longer windows (e.g., 400–1000 ms; MacNamara et al. 2016; MacNamara and Proudfit 2014; Weinberg and Hajcak 2011). Our main and supplementary findings indicate that GAD symptoms may relate to the LPP across the first second of processing. Our findings are consistent with theories that describe GAD as linked to heightened reactivity to negative stimuli (Mennin et al. 2002). Although the neural correlates of GAD are heterogeneous (Goossen et al. 2019), our findings add to an emerging body of work implicating heightened LPP response to negative (vs. neutral) images as a neural correlate of GAD symptoms.

Our findings complement an emerging body of work examining the LPP and AS, especially recent evidence that AS is related to a greater LPP response to images depicting anxious arousal (Allan et al. 2019; Hamrick et al. 2024). Our data indicate that AS is also related to the LPP response to negative images commonly used to study anxiety pathology. Future research may continue to examine how AS relates to LPP responses to various stimuli and even the development of anxiety vulnerability. For example, a study of 5–7 year olds found that the LPP response to negative images was related to parent sensitivity to their child's anxiety, but only when the parent was in the room (Day et al. 2020). More research on AS and the LPP is needed to understand how context, particularly parent–child relationships, may modulate attention to negative stimuli.

Our results may be understood in the context of literature examining how IU relates to LPP responses to negative images. Stimuli conditioned with shock or imagined negative scenes have been more commonly studied to study IU than negative and neutral images. At first glance, our findings appear inconsistent with those of MacNamara et al. (2018), who found that IU was related to a smaller LPP when participants were instructed to imagine a negative scene described to them. But their results may be reconciled with ours. It may be speculated that people high in IU avoid imagining details of a negative scene, resulting in a lower LPP. But attention to negative images that are present on a screen may be less easily suppressed or avoided than attention to imagined details. Another set of findings from a study of fear generalization by Nelson et al. (2015) aligns more clearly with ours. IU was associated with a smaller PCA‐derived LPP factor response to generalization stimuli (i.e., stimuli resembling to varying degrees a conditioned stimulus that preceded electric shock). These data may be interpreted as indicating that IU was associated with less fear generalization (i.e., better distinction between threat and safety cues). Similarly, Bauer et al. (2020) found that IU was associated with a larger LPP difference between threat and safety cues during extinction. Aligning with these data, our findings may be interpreted as indicating greater distinction between attention given to negative and neutral images, indicated by a larger LPP difference. Our findings especially complement those of Gole et al. (2012) who found that participants high in IU had a larger LPP response to negative images when accurately cued than when they were ambiguously cued (i.e., unpredictable). Differing from these findings, but consistent with ours, Wiese et al. (2023) found IU to relate to a larger LPP response to negative images regardless of being cued accurately or ambiguously. An analysis of whether IU was associated with the response to accurately cued negative images was not reported, likely because the ANOVA indicated a superordinate interaction, but our findings support this association.

Our data were tested extensively to address limitations of LPP measurement and analysis. The early LPP responses had good reliability as estimated from standard measurement errors. We focused on the early residual‐based LPP, which had acceptable reliability, whereas the difference score did not. The results were consistent regardless of using a frequentist or Bayesian approach or using a residual‐based or difference score to measure the LPP. The results of the alternate approaches are in the Supporting Information S1 to inform researchers employing different analytical approaches.

Some limitations to the study should be noted. First, we used a convenience sample of undergraduate students. Although almost a third of our sample scored at or above the cutoff recommended for GAD, the sample limits generalization with respect to clinical groups or the broader population. However, this aspect of our design does facilitate comparison between our studies and prior work on the LPP, much of which used undergraduate student samples. Second, studying GAD symptoms dimensionally rather than categorically limits conclusions about GAD as a diagnostic category. But our approach complements studies of diagnosed GAD (e.g., MacNamara and Hajcak 2010) and is consistent with models that emphasize the dimensional nature of psychopathology (Cuthbert 2014; Kotov et al. 2017). Third, the cross‐sectional design prevents causal interpretations, including the role of AS and IU as mechanisms. Sensitivity analyses contained in the Supporting Information S1 suggest that it may be worthwhile to test the findings in a longitudinal design. Nevertheless, the indirect effects should not be interpreted as evidence for causal mediation. Fourth, the use of static images selected from a standardized database prioritized internal validity, but the relation between the LPP and anxiety may be most pronounced when stimuli are personally relevant (Botelho et al. 2023) and more ecologically valid (Stolz et al. 2019). Fifth, and finally, our paradigm did not examine important factors explored in prior work, including predictability, cognitive load, emotion regulation, and fear learning/extinction processes. Nevertheless, our results add to the basis on which cognitive factors, such as AS and IU, may be further studied as mechanisms of GAD.

## Author Contributions

**Matt R. Judah:** conceptualization, formal analysis, methodology, project administration, resources, software, supervision, visualization, writing – original draft. **Hannah C. Hamrick:** data curation, investigation, software, writing – original draft, writing – review and editing. **Morgan S. Middlebrooks:** writing – original draft, writing – review and editing. **Benjamin Swanson:** software, visualization, writing – review and editing. **Grant S. Shields:** conceptualization, methodology, software, visualization, writing – original draft, writing – review and editing.

## Conflicts of Interest

The authors declare no conflicts of interest.

## Supporting information


Data S1.

## Data Availability

The data that support the findings of this study are available from the corresponding author upon reasonable request.

## Endnotes

^1^
IAPS image numbers. Negative: 2095, 2683, 2688, 2730, 2800, 2900, 3030, 3060, 3100, 3101, 3120, 3170, 3180, 3181, 3191, 3220, 3230, 3261, 3266, 3350, 3500, 3530, 6212, 6213, 6260, 6350, 6360, 6510, 6560, 7380, 9040, 9050, 9250, 9252, 9253, 9300, 9321, 9340, 9400, 9410, 9419, 9420, 9428, 9435, 9500, 9520, 9530, 9560, 9570, 9571, 9600, 9800, 9810, 9900, 9901, 9903, 9910, 9921, 9922, 9925. Neutral: 2037, 2038, 2102, 2190, 2191, 2200, 2210, 2214, 2215, 2221, 2271, 2272, 2280, 2302, 2381, 2383, 2385, 2393, 2396, 2397,2440, 2441, 2480, 2493, 2495, 2499, 2512, 2514, 2516, 2570, 2595, 2749, 2840, 2850, 2870, 2880, 2890, 4000, 5390, 5410, 5471, 5510, 5726, 5731, 5740, 5800, 5870, 5875, 7002, 7004, 7025, 7100, 7130, 7234, 7290, 7493, 7550, 7590, 7700, 9070.

^2^
Boxplots revealed one ASI‐3 outlier and two IUS‐SF outliers. Analyses were repeated with outliers winsorized at the 95th percentile. The direction and significance of the results remained unchanged. Reported results used untransformed variables.
