# Supplementary material for: Anxiety Sensitivity and Intolerance of Uncertainty Uniquely Explain the Association of the Late Positive Potential With Generalized Anxiety Disorder Symptoms
Source: Psychophysiology. 2025 Apr 3;62(4):e70044. doi: 10.1111/psyp.70044 (PMC11966597; doi:10.1111/psyp.70044)
Supplement: Supplementary file 1 — Data S1. [file PSYP-62-e70044-s001.docx]

**Supplementary Materials**

**Sensitivity Analysis**

To address potential bias in the cross-sectional mediation model parameters and inform the viability of a future test in a longitudinal design, we used Georgeson and colleagues’ (2023) sensitivity analysis procedure. The procedure simulates a two-wave mediation model with phantom wave 1 mediator and outcome variables with fixed variances. The indirect effect is then tested across a range of values for the autoregressive and cross-lagged paths. In our analysis, we estimated autoregressive correlations for both the mediator (phantom mediator at T1 predicting observed mediator) and outcome (phantom GAD symptoms at T1 predicting observed GAD symptoms) at correlations between 0 and .6 at intervals of .1. This is a reasonable range suggested by Georgeson and colleagues as these are partial correlations. Further, we tested cross-lagged paths (phantom mediator predicting observed GAD symptoms and phantom GAD symptoms predicting observed mediator) at correlations from -.6 to .6 at intervals of .1. In total, 53 simulations were conducted. The correlation between the phantom mediator at T1 and the phantom outcome at t1 was fixed to .56 based on our observed bi-variate correlation between anxiety sensitivity and anxiety. The simulation results revealed that the indirect effect remained statistically significant in 52 of 53 simulations (see Supplementary Materials Figure 1).

**Residual-based LPP 700 – 1000 ms**

The residual-based LPP from 700 – 1000 ms was associated with greater AS, *a1* = .31 [.10, .51] and greater IU, *a2* = .33 [.12, .53]. Higher AS predicted higher GAD-7 scores, *b1* = .36 [.16, .55]. Higher IU also predicted higher GAD-7 scores, *b2* = .29 [.10, .48]. There was an indirect effect of the LPP on GAD-7 scores through AS (*a1b1* = .11 [.04, .20]) and through IU (*a2b2* = .09 [.03, .19]). The indirect effects contrast, .01 [-.10, .13], showed that the indirect effects did not differ. The total effect, *c* = .39 [.20, .59], accounted for 15.58% of the variance in GAD-7 scores. There was a direct effect of the LPP on GAD-7 scores, *c′* = .19, [.01, .37].

**Residual-based LPP 1000 – 2000 ms**

The residual-based LPP from 1000 – 2000 ms was not associated with AS, *a1* = .09 [-.13, .30] or IU, *a2* = -.01 [-.22, .21]. Higher AS predicted higher GAD-7 scores, *b1* = .41 [.21, .60]. Higher IU also predicted higher GAD-7 scores, *b2* = .33 [.13, .52]. There was no indirect effect of the LPP on GAD-7 scores through AS (*a1b1* = .04 [-.04, .14]) or IU (*a2b2* = -.00 [-.06, .08]). There was no total effect, *c* = -.08 [-.30, .13], *R*^2^ = .01, or direct effect of the LPP on GAD-7 scores, *c′* = -.11, [-.28, .05].

**Difference LPP 400 – 700 ms**

The difference LPP (negative – neutral) was associated with greater AS, *a1* = .24 [.03, .45], and greater IU, *a2* = .24 [.03, .45]. Higher AS predicted higher GAD-7 scores, *b1* = .37 [.18, .57]. Higher IU also predicted higher GAD-7 scores, *b2* = .32 [.12, .51]. There was an indirect effect of the difference LPP on GAD-7 scores through AS (*a1b1* = .09 [.01, .19]) and through IU (*a2b2* = .07 [.01, .16]). The indirect effects contrast, .01 [-.09, .12], showed that the indirect effects did not differ. The total effect, *c* = .29 [.08, .50], accounted for 8.36% of the variance in GAD-7 scores. There was no direct effect of the difference LPP on GAD-7 scores, *c′* = .13, [-.05, .30].

**Difference LPP 700 – 1000 ms**

The difference LPP from 700-1000 ms was associated with greater AS, *a1* = .30 [.10, .51] and greater IU, *a2* = .32 [.11, .52]. Higher AS predicted higher GAD-7 scores, *b1* = .36 [.16, .55]. Higher IU also predicted higher GAD-7 scores, *b2* = .29 [.10, .49]. There was an indirect effect of the LPP on GAD-7 scores through AS (*a1b1* = .11 [.03, .21]) and through IU (*a2b2* = .09 [.03, .19]). The indirect effects contrast, .01 [-.10, .13], showed that the indirect effects did not differ. The total effect, *c* = .20 [.09, .34], accounted for 14.60% of the variance in GAD-7 scores. There was a direct effect of the LPP on GAD-7 scores, *c′* = .18, [.00, .36].

**Difference LPP 1000 – 2000 ms**

The difference LPP from 1000 – 2000 ms was not associated with AS, *a1* = .11 [-.21, .21] or IU, *a2* = -.05 [-.26, .17]. Higher AS predicted higher GAD-7 scores, *b1* = .41 [.21, .60]. Higher IU also predicted higher GAD-7 scores, *b2* = .32 [.13, .52]. There was no indirect effect of the LPP on GAD-7 scores through AS (*a1b1* = .05 [-.03, .16]) or IU (*a2b2* = -.02 [-.08, .06]). There was no total effect, *c* = -.06 [-.28, .15], *R*^2^ = .00, or direct effect of the LPP on GAD-7 scores, *c′* = -.09, [-.26, .08].

**Residual-based LPP 400 – 700 ms Predicting Social Anxiety**

The residual-based LPP from 400-700 ms was associated with greater AS, *a1* = .23 [.02, .44] and greater IU, *a2* = .24 [.03, .45]. Higher AS predicted higher SIAS-6 scores, *b1* = .35 [.16, .55]. Higher IU also predicted higher SIAS-6 scores, *b2* = .38 [.19, .58]. There was an indirect effect of the LPP on SIAS-6 scores through AS (*a1b1* = .08 [.01, .19]) and through IU (*a2b2* = .09 [.01, .21]). The indirect effects contrast, -.01 [-.13, .11], showed that the indirect effects did not differ. There was no total effect, *c* = .19 [-.03, .40] or direct effect of the LPP on SIAS-6 scores, *c′* = .01, [-.16, .19].

**Residual-based LPP 400 – 700 ms Predicting Depression**

The residual-based LPP from 400-700 ms was associated with greater AS, *a1* = .23 [.02, .44] and greater IU, *a2* = .24 [.03, .45]. Higher AS predicted higher PHQ-9 scores, *b1* = .38 [.19, .57]. Higher IU also predicted higher PHQ-9 scores, *b2* = .35 [.16, .54]. There was an indirect effect of the LPP on PHQ-9 scores through AS (*a1b1* = .09 [.01, .21]) and through IU (*a2b2* = .09 [.01, .19]). The indirect effects contrast, .00 [-.11, .13], showed that the indirect effects did not differ. The total effect, *c* = .25 [.04, .46], accounted for 6.29% of the variance in PHQ-9 scores. There was not a direct effect of the LPP on PHQ-9 scores, *c′* = .08, [-.09, .25].


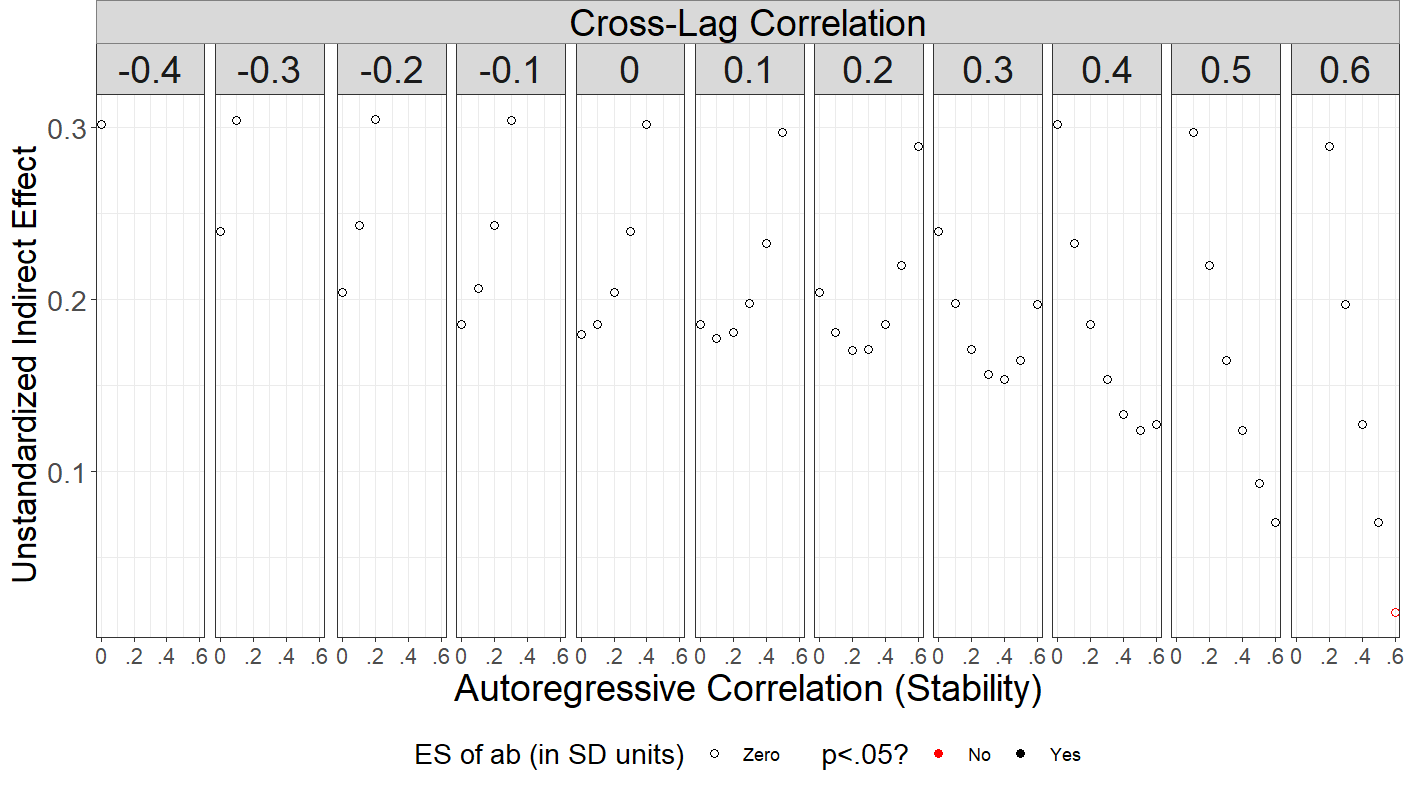
*Figure 1.* Sensitivity analysis using phantom variables (previous wave mediator and outcome variables) to estimate indirect effects at various autoregressive and cross-lagged correlations.
